# Analyzing the European institutional response to ethical and regulatory challenges of artificial intelligence in addressing discriminatory bias

**DOI:** 10.3389/frai.2024.1393259

**Published:** 2024-06-25

**Authors:** Pablo Cerezo-Martínez, Alejandro Nicolás-Sánchez, Francisco J. Castro-Toledo

**Affiliations:** ^1^Plus Ethics, Elche, Spain; ^2^Ethical and Legal Plus, Elche, Spain

**Keywords:** artificial intelligence, discriminatory bias, Europe, ethics, regulatory framework

## Abstract

The European Union and some of its institutions have taken significant steps to address the challenges posed by the development and use of Artificial Intelligence (AI) in various contexts. The ubiquity of AI applications in everyday life, affecting both citizens and professionals, has made AI a common topic of discussion. However, as is evident from the documents analyzed here, concerns have been raised about the possible negative social consequences of AI, in particular discriminatory bias, making it a particularly relevant issue if people-centred, rights-based AI is to be implemented. This article aims to examine the challenges of defining, identifying and mitigating discriminatory bias in AI systems from two perspectives: (1) to conduct an ethical and normative review of European Commission documents from the last 8 years (from GDPR to AI Act regulation); and (2) to expose recommendations for key stakeholders, including designers, end-users and public authorities, to minimize/mitigate this risk. The document review was carried out on 21 EU regulatory and ethical guidelines in the field of AI, from which 152 measures were extracted, differentiated between design, governance and organizational measures. It has also been observed that there is no clear conceptual framework on the issue at the European level, showing a clear problem in providing definitions of algorithmic bias and discrimination, but not in assessing their potential negative impact on individuals. Secondly, these gaps may affect the concreteness and detail of the possible mitigation/minimization measures proposed and, subsequently, their application in different contexts. Finally, the last section of this paper presents a brief discussion and conclusions on possible issues related to the implementation of the measures extracted and certain limitations of the study.

## 1 Introduction

The European Union and its institutions have made numerous efforts to identify and address the challenges posed by the development and implementation of artificial intelligence (AI) in the various contexts in which it is intended to be used. In this regard, since March of 2018 a number of relevant milestones have been remarked by European Commission such as: Launch of the European AI Alliance, set up of the high-level expert group on AI, the White paper on AI: a European approach to excellence and trust, the Proposal for a regulation laying down harmonized rules on AI^1^ or the Proposal for an AI liability directive among others.^2^

This has highlighted the cross-cutting nature of these tools, which can be applied in virtually all contexts of daily life, both for citizens and for professionals in different fields; it is this proliferation of applications and the gradual improvement of tools, making them more powerful and efficient, that has led to AI becoming a topic of common discussion. However, in parallel with this progress, institutional voices have become increasingly vocal about the potential negative impact of these tools, including the issue of discriminatory bias. Generally speaking, the AI risks that have raised the most concern include the following: (1) AI algorithms can perpetuate and amplify existing biases in the data, leading to discriminatory outcomes (bias and discrimination) (Mayson, 2019); (2) many AI models, especially the more advanced ones, are ‘black boxes’ that provide little or no insight into how they reach their conclusions (lack of transparency) (Ribeiro et al., 2016; Molnar, 2022); (3) the use of personal data in AI raises concerns about privacy and consent (ethical and privacy issues) (Véliz, 2020; Richards, 2021); (4) data quality is critical to AI performance, and faulty data can lead to erroneous results (data quality dependency) (Byabazaire et al., 2020); (5) AI-driven automation can displace human jobs, creating economic and social challenges (unemployment and job displacement) (Frey and Osborne, 2017; Acemoglu and Restrepo, 2019; Acemoglu et al., 2022) (6) AI can be used for harmful purposes, and AI systems are vulnerable to attack and manipulation (security and misuse) (Brundage et al., 2018).

In Europe, several concrete examples of bias AI have recently been identified. To name just a few of the most recent, in the field of recruitment, for example, AI tools were used that turned out to be biased against women. This happened when the AI was based on CVs submitted over the last 10 years, most of which belonged to men, leading the algorithm to favor men over women (Dastin, 2018). This trend of using AI in recruitment is expected to continue in 2024, although measures are also being taken to reduce the risk of bias.^3^ Another recent example of AI bias in Europe is the scandal in the Netherlands, where the government used an algorithm to predict who was likely to fraudulently claim child benefit. Without any evidence of fraud, the tax authorities forced 26,000 parents, targeting dual nationals and ethnic minorities, to pay back tens of thousands of euros with no right of appeal. The Dutch Data Protection Authority found the tax authorities’ methods to be ‘discriminatory’ (Henley, 2021; Heikkilä, 2022).

The aim of this article is to address the challenges of defining, identifying and minimising discriminatory bias in AI systems within a European scope (rather guarantee-based, from an international comparative perspective) from a double point of view: (a) based on an ethical and normative review of the reference documents published by European public bodies (and its different working groups) over the last 8 years, and (b) with an applied purpose for the main stakeholders (designers, consumers, public authorities, etc.). To achieve this, the following content structure is proposed: in the first section, the concept of algorithmic discrimination will be introduced from a multidisciplinary perspective; in the second section, the main results of the quantitative and qualitative comprehensive and analytical review of the approach to the issue of discriminatory bias in the main European regulatory instruments and recommendations related to the design, development, implementation and use of AI systems will be presented; and finally, a third section will aim at outlining and categorising the recommendations to minimize and mitigate this risk. In short, this proposal makes it possible to describe the state of the art of the European ethical and legal framework for responding to this phenomenon in a feasible and workable way.

## 2 Discriminatory bias in AI

### 2.1 Scope and impact of discriminatory bias in AI

Throughout the literature reviewed, comprehensive delineations of the phenomenon known as algorithmic discrimination are infrequent.^4^ Instead, comprehension arises predominantly from the ramifications it engenders, especially those entailing inequitable or disparate decision-making among individuals without apparent basis and due to the existence of discriminatory biases (O’Neil, 2016; Buolamwini and Gebru, 2018; Eubanks, 2018; Noble, 2018).^5^ Consequently, manifestations of discriminatory trends stemming from the deployment of AI tools manifest across diverse domains, including those previously elucidated, along with others necessitating the use of such tools, such as the medical realm (Rajkomar et al., 2018; Obermeyer et al., 2019) and the economic sphere (Mendes and Mattiuzzo, 2022). Such manifestations harbor the potential to result in uneven treatment predicated on factors encompassing race, gender, ethnicity, and more. And, similarly, algorithmic discrimination can also occur when “a computerized model makes a decision or a prediction that has the unintended consequence of denying opportunities or benefits more frequently to members of a protected class than to an unprotected control set” (Brownstein, 2022).

Taking into account the above issues, it is possible to understand algorithmic discrimination as the harmful unjustified consequences experienced by individuals as a result of outcomes generated by AI tools that operate with specific algorithms. Similar definitions can be found in (Brownstein, 2022) or (The White House, n.d.) when it is stated that: “Algorithmic discrimination” occurs when automated systems contribute to unjustified different treatment or impacts disfavoring people based on their race, color, ethnicity, sex (including pregnancy, childbirth, and related medical conditions, gender identity, intersex status, and sexual orientation), religion, age, national origin, disability, veteran status, genetic information, or any other classification protected by law. These patterns of discrimination are significant. The need for extensive data collection to support labeling, profiling, recognition or decision making driven by AI algorithms, and the resulting consequences, has sparked a profound debate about the potential impact on individuals (Cuquet and Fensel, 2018; Zuboff, 2019; Véliz, 2020). For example, when examining any of these tools, algorithmic profiling often emerges as a source of discrimination (Eubanks, 2018; Noble, 2018; Mann and Matzner, 2019), along with the phenomenon known as the chilling effect (FRA, 2019a; Büchi et al., 2020). The chilling effect embodies altered behavioral patterns resulting from fear of surveillance: a form of self-censorship in which individuals strive to avoid negative external perceptions or present an overly positive image. These algorithms work by identifying correlations and making predictions about group-level behavior, with groups (or profiles) being continually redefined by the algorithm (Zarsky, 2013). Understanding of individuals, whether dynamic or static, is based on associations with others identified by the algorithm, rather than being rooted in actual behavior (Newell and Marabelli, 2015). As a result, profiling often shapes decisions about individuals through group-derived information (Danna and Gandy, 2002; Malek, 2022), inadvertently leading to the creation of databases that facilitate discrimination (de Vries, 2010). Furthermore, as will be elucidated in the following sections, discriminatory analyses rooted in various types of prejudice can foster self-fulfilling prophecies, misuses, stigmatising marginalized groups and impeding their autonomy and social participation (Macnish, 2012; Leese, 2014; Castro-Toledo, 2022; Cerezo-Martínez et al., 2022).

On the other hand, eminent challenges plaguing AI tools that rely on data training focus on the origin of the data. A significant proportion of algorithmic discrimination arises from non-random patterns within data, derived from pre-existing biased databases. This includes imbalances in age, gender, ethnicity and other relevant risk factors, as well as outdated or inaccurate data. Similar challenges may arise from analytical shortcomings due to insufficient data or other reasons. However, these challenges may be no more severe than those encountered when human decision making is carried out without computer systems (Bechtel et al., 2017). Nevertheless, there is still a palpable reality: significant ethical challenges remain, stemming from the lack of a well-defined and operational concept of algorithmic fairness. Some characterize this as the need for algorithmic results to be equally accurate, or to produce an equal number of false positives and false negatives for members of different social groups (Hellman, 2020).

Concerns have also arisen about whether the effectiveness and validity of these tools vary according to the gender of the individual being assessed. For example, the Level of Service Inventory-Revised (LSI-R), which is widely used in the United States, has been criticized for its specificity in predicting antisocial male behavior, with a weaker predictive ability for female behavior. This has led to calls for the development of more gender-specific instruments (Smith et al., 2009; Donnelly and Stapleton, 2022; Olver and Stockdale, 2022) and for gender-sensitive approaches to misconduct risk assessment (Hannah-Moffat, 2009). Contrary findings have also been reported, such as the gender-neutrality of the DRAOR tool as found by Scanlan et al. (2020). Therefore, gender dynamics in risk prediction warrant a comprehensive review that addresses the neutrality of tools in this regard.

### 2.2 Some Key normative-ethical bases for the European response to AI shortcomings

In the previous section, the various reasons for identifying the negative and problematic discriminatory effects of the use of AI tools are manifold and far from being fully addressed. In this regard, the gradual advancement of AI functionalities has led to growing concerns about the ethical, legal and social consequences of their design, development and deployment. These concerns have spurred the creation of numerous ethical and regulatory frameworks in the European context, with the main objective of defining, analysing, minimising and mitigating the potential impacts that AI tools may have in different application contexts.

An examination of the European normative-ethical framework reveals a common consensus, despite possible differences in the interpretation of concepts. This consensus emphasises that AI-driven tools should be developed, deployed and used in accordance with a set of principles, both at an ethical level, including fairness, accountability or transparency, among others; and at a legal level, such as respecting fundamental rights through non-discrimination and the right to privacy. This ethical and legal approach aims to establish a unified European framework for the development of AI. In other words, the incorporation of AI in various sensitive areas has potential implications for fundamental rights and civil liberties if clear limits are not set for its use (FRA, 2018a,b,c). Therefore, an AI system that complies with a set of ethical and legal standards underpins the entire rationale of European research and aspirations, and AI designers should respect fundamental European ethical values such as justice, fairness, privacy or transparency for different ethical and normative reasons. Here only a few will be mentioned. First, because ethical AI can help avoid the emergence and spread of biases that lead to discriminatory or stigmatising practices. Training with data that is biased by race, gender, age or other factors can be key to perpetuating and reinforcing existing prejudices and inequalities. To mitigate this problem, it is precisely necessary to develop AI that is unbiased and takes into account principles such as diversity, universality or plurality (AI HLEG, 2019; FRA, 2019b; STOA and EPRS, 2022). Second, ethical AI can contribute to public benefit. AI has the potential to address many global challenges. However, if used unilaterally or against the shared values of society as a whole, it can have potential consequences for both users and those affected by it (AI HLEG, 2019; FRA, 2022). It is therefore prudent to develop AI that contributes to the well-being of society as a whole, not just some groups. Third, ethical AI can foster trust in technology and innovation. The trust that developers, end-users and citizens can place in AI systems is fundamental to their effective and safe use (AI HLEG, 2019; FRA, 2020; STOA and EPRS, 2020). If key actors involved in the development, implementation and use of AI do not trust it, they may be reluctant to use it, which could limit its operability. It is therefore necessary to develop AI that is transparent, responsible, explainable and accountable. Fourth, ethical AI can be safer, more accurate and more reliable. AI tools can be subject to errors, third-party attacks and manipulation, which can have serious consequences for both users and those affected by them. By developing AI that respects ethical standards, more robust security, privacy, accuracy, tuning and monitoring measures can be implemented, which can reduce the risk of security incidents and improve the reliability of the technology (AI HLEG, 2019; FRA, 2019b).

In addition, issues in relation to algorithmic discrimination have also been the focus of attention in some relevant European guidelines, such as “The Ethics of artificial intelligence: Issues and initiatives” by STOA in 2020, the “Ethics Guidelines for Trustworthy AI” by EC experts in 2019, and its modeling by the “Assessment List for Trustworthy AI” (ALTAI), among others. The existence of potential biases in AI tools is a serious concern and a source of analysis and discussion on their definition, impact and strategies to minimize and mitigate them. Likewise, it does not seem possible to limit the existence of these biases to a specific phase of the overall development of the tools, but rather it is a cross-cutting problem that can be present and affect both the designers and the end users of these tools throughout the process. It is precisely this approach that is reflected in the concept of Ethics by Design, also developed in the European reference framework by the EC in the document, “Ethics by Design and Ethics of Use Approaches for Artificial Intelligence” (2021a). Following the definition given for this approach: “Ethics by Design is an approach that can be used to ensure that ethical requirements are properly addressed during the development of an AI system or technique” (European Commission, 2021a, p.11) and that: “Ethics by Design aims to prevent ethical issues from arising in the first place by addressing them during the development phase, rather than trying to fix them later in the process” (European Commission, 2021a, p.12).

Regardless of the methodologies used to carry out this monitoring, which may vary depending on the starting conditions or expected uses, the central point is that the focus should not be exclusively on mitigation measures to address the impacts that may be caused by the misuse of AI tools. Instead, it may be more beneficial to adopt an approach based on prevention of problems that are already recognized as existing and having a potential impact on people. For example, a detailed analysis of potential risks in the form of biases that developers may face in the early stages of ideation and development of AI tools may ultimately lead to a reduction in potential harmful impacts on people. Or, for example, it could be precautionary to analyze the databases used for algorithmic decision-making at the source, as also stated in the STOA 2022 report, “Auditing the quality of datasets used in algorithmic decision-making systems,” which, as has been pointed out, can be a clear source of bias from the design of the data, its collection, processing and maintenance.

In this sense, the establishment of a permanent monitoring task, covering all phases from design and implementation to end-user use, could significantly improve the development of these AI tools. These European guidelines include recommendations and strategies, both at the ethical and legal level, to achieve reliable AI and also to avoid possible biases arising from its development, management or use. In any case, although these recommendations and practices aimed at analysing the impact and establishing recommendations to minimize and mitigate bias are present in all European documents, ALTAI is currently the guideline that establishes the clearest and most concise way to address them, as it sets out specific questions regarding their possible impact and the specific actions to deal with them, in terms of avoiding unfair bias, accessibility and universal design, and stakeholder participation.

To sum up, in the European context, the wide range of concerns about the ethical, legal and social implications of AI has led to the development of ethical and regulatory frameworks. These frameworks aim to ensure that AI adheres to ethical principles, respects fundamental rights and addresses potential biases throughout its life cycle. However, their correct assimilation and consequently their correct implementation by all interested parties has been complicated by both the growing number of European guidance attempts and their dispersion over time. In response to this complex context of institutional guidelines, their content will be analyzed in the following section.

## 3 Mapping of the main European ethical and normative AI guidelines

### 3.1 Methodology applied and AI (or related) guidelines assessed

This review was carried out on the basis of 21 European guidelines issued by different public institutions. Although this is not a systematic review as defined by the PRISMA recommendations (Page et al., 2021), the documents analyzed meet the following inclusion criteria:

1. Published by a public European public institution, such as: European Union Agency for Fundamental Rights (FRA), Panel for the Future of Science and Technology (STOA) & European Parliamentary Research Service (EPRS) (2019), European Commission (EC) (European Commission, 2018, 2020a, 2020b, 2021b), High-Level Expert Group on Artificial Intelligence (AI HLEG), Council of the European Union (CoEU) (Council of the European Union, 2020), Council of Europe (CoE) (Council of Europe, 2019, 2020) and European Parliament (EP) (European Parliament, 2017, 2023).
2. Date, establishing a timeline that oscillates between 2016, with the first document considered (GDPR), and 2023, with the last update of the Artificial Intelligence Act (AI Act) (European Commission, 2024). It starts with the GDPR, as this relevant legal framework serves as a starting point for considering and preventing risks that may arise from AI tools handling personal data in terms of individuals’ rights.
3. Openly accessible documents from different disciplines and sources that mark the path of technical, social, ethical and legislative development of AI, with a particular focus on those that contain references to the concept of unfair and discriminatory algorithmic bias. In order to provide a more holistic and detailed understanding of the complex issues at stake, a wide range of document types (e.g., policies, reports, legislation, recommendations) were taken into account, which would be much more complex to carry out in a systematic review due to the need for strict criteria. Due to the institutional nature of the documents analyzed, all of them were collected and consulted via the official websites of the public institutions identified in criterion (1).

Table 1 provides a chronology of the main ethical and regulatory guidelines that marked the progress of AI in Europe from 2016 to 2024 included in the analyses.

**Table 1:** Chronology of the main European ethical and normative AI guidelines analyzed.

Date^1^	Document	Institution
2024	Artificial Intelligence Act^2^	EC, EP, CoEU
2022	Bias in algorithms – artificial intelligence and discrimination	FRA
	Auditing the quality of datasets used in algorithmic decision-making systems	STOA & EPRS.
2021	Ethics by design and ethics of use approaches for artificial intelligence	EC
	Algorithmic discrimination in Europe. Challenges and opportunities for gender equality and non-discrimination law	EC
2020	Getting the future right. Artificial intelligence and fundamental rights	FRA
	Presidency conclusions. The charter of fundamental rights in the context of artificial intelligence and digital change	CoEU
	Assessment list for trustworthy AI (ALTAI)	EC (AI HLEG)
	Recommendation CM/Rec (2020) 1 of the committee of minister to member States on the human rights impacts of algorithmic systems	CoE
	The ethics of artificial intelligence: issues and initiatives	STOA & EPRS
	Gender equality strategy 2020–2025	EC
	White paper on artificial intelligence -A European approach to excellence and trust	EC
2019	Data quality and artificial intelligence -mitigating bias and error to protect fundamental rights-	FRA
	Unboxing artificial intelligence: 10 steps to protect human rights	CoE
	Ethics guidelines for trustworthy AI (HLEG)	EC (AI HLEG)
	Understanding algorithmic decision-making: opportunities and challenges	STOA & EPRS
2018	Preventing unlawful profiling today and in the future: a guide	FRA
	BigData: discrimination in data-supported decision making	FRA
	European AI Strategy	EC
2017	Fundamental rights implications of big data	EP
2016	General data protection regulation (GDPR)	EP & CoEU

^1^This date refers to the last update of the analyzed document.

^2^The latest version of this document refers to 19/04/2024 and corrects drafting and numbering errors presents in previous drafts. This document is called “Corrigendum to the position of the European Parliament adopted at first reading on March 13, 2024, with a view to the adoption of Regulation (EU) 2024” (European Parliament, 2024). Available at: https://www.europarl.europa.eu/doceo/document/TA-9-2024-0138-FNL-COR01_EN.pdf.

On the other hand, the following variables were systematically evaluated in each of the included document and answered in a dichotomous way (i.e., yes or no):

- Whether or not they provide a definition of the term bias or algorithmic bias,
- Whether they establish recommendations or measures to mitigate and minimize algorithmic bias;
- Whether or not they are in force. In the case of reports that cannot be directly implemented, the answer “NO” has been chosen to indicate that these are guidelines that can be used for analysis but, strictly speaking, are documents whose content is not mandatory.

### 3.2 General overview

**Figure 1:**
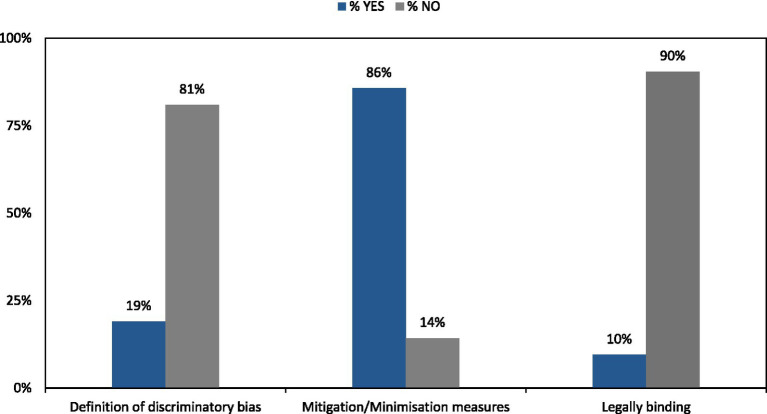
Quantitative summary of the analysis of European documents regarding the ethical and regulatory framework of AI.

The review of European guidelines on AI reveals some key findings (Table 2; Figure 1). While the concept of bias is seldom explicitly mentioned, the documents acknowledge its origins and multifaceted impacts—social, legal, and ethical. Only 19% of the documents directly address bias, with 81% omitting it. Furthermore, 86% of the documents propose various measures to mitigate bias, with 14% lacking such measures. Notably, only 10% of the analyzed documents and regulations are legally binding, while the remaining 90% are non-binding as most are informative reports, briefings, or studies offering guidance and recommendations, rather than binding directives for member States.

**Table 2:** Qualitative summary of European ethical and normative AI guidelines.

Reference	Date of publication (dd/mm/yy)	Scope of the document	Definition of discriminatory bias	Mitigation / minimisation measures	Legally binding
Artificial Intelligence Act (AI Act)	21/04/2021 approved (13/03/2024)	It’s a proposed European law on artificial intelligence (AI). The law assigns applications of AI to three risk categories. First, applications and systems that create an unacceptable risk. Second, high-risk applications. Lastly, applications not explicitly banned or listed as high-risk are largely left unregulated.	No	Yes	Yes
Bias in Algorithms – Artificial Intelligence and Discrimination	8/12/2022	The report looks at the use of artificial intelligence in predictive policing and offensive speech detection. It demonstrates how bias in algorithms appears, can amplify over time and affect people’s lives, potentially leading to discrimination. It corroborates the need for more comprehensive and thorough assessments of algorithms in terms of bias before such algorithms are used for decision-making that can have an impact on people.	Yes	Yes	No
Auditing the quality of datasets used in algorithmic decision-making systems	25/07/2022	This study begins by providing an overview of biases in the context of artificial intelligence, and more specifically to machine-learning applications. The second part is devoted to the analysis of biases from a legal point of view. The analysis shows that shortcomings in this area call for the implementation of additional regulatory tools to adequately address the issue of bias. Finally, this study puts forward several policy options in response to the challenges identified.	No	Yes	No
Ethics by design and ethics of use approaches for artificial intelligence	25/11/2021	Offers guidance for adopting an ethically-focused approach while designing, developing, and deploying and/or using AI based solutions. It explains the ethical principles which AI systems must support and discusses the key characteristics that an AI-based system/ applications must have in order to preserve and promote.	No	Yes	No
Algorithmic discrimination in Europe. Challenges and opportunities for gender equality and non-discrimination law	10/03/2021	This report investigates how algorithmic discrimination challenges the set of legal guarantees put in place in Europe to combat discrimination and ensure equal treatment. More specifically, it examines whether and how the current gender equality and non-discrimination legislative framework in place in the EU can adequately capture and redress algorithmic discrimination.	Yes	Yes	No
Getting the future right. Artificial intelligence and fundamental rights	14/12/2020	This report presents concrete examples of how companies and public administrations in the EU are using, or trying to use, AI. It focuses on four core areas – social benefits, predictive policing, health services and targeted advertising.	No	No	No
Presidency conclusions -the charter of fundamental rights in the context of artificial intelligence and digital change	21/10/2020	Conclusions on the charter of fundamental rights in the context of artificial intelligence and digital change. These conclusions are designed to anchor the EU’s fundamental rights and values in the age of digitalization, foster the EU’s digital sovereignty and actively contribute to the global debate on the use of artificial intelligence with a view to shaping the international framework.	No	Yes	No
Assessment list for trustworthy AI (ALTAI)	17/07/2020	Through the Assessment List for Trustworthy AI (ALTAI), AI principles are translated into an accessible and dynamic checklist that guides developers and deployers of AI in implementing such principles in practice. ALTAI will help to ensure that users benefit from AI without being exposed to unnecessary risks by indicating a set of concrete steps for self-assessment.	Yes	Yes	No
Recommendation CM/Rec(2020)1 of the committee of ministers to member States on the human rights impacts of algorithmic systems	8/04/2020	Underlying that member States must ensure that any design, development and ongoing deployment of algorithmic systems occur in compliance with human rights and fundamental freedoms, which are universal, indivisible, interdependent and interrelated, with a view to amplifying positive effects and preventing or minimising possible adverse effects.	No	Yes	No
The ethics of artificial intelligence: Issues and initiatives	11/03/2020	The study deals with the ethical implications and moral questions that arise from the development and implementation of artificial intelligence (AI) technologies. It also reviews the guidelines and frameworks that countries and regions around the world have created to address these. It presents a comparison between the current main frameworks and the main ethical issues, and highlights gaps around mechanisms of fair benefit sharing; assigning of responsibility; exploitation of workers; energy demands in the context of environmental and climate changes; and more complex and less certain implications of AI, such as those regarding human relationships.	No	Yes	No
Gender equality strategy 2020–2025	5/03/2020	This Gender Equality Strategy frames the European Commission’s work on gender equality and sets out the policy objectives and key actions for the 2020–2025 period.	No	No	No
White paper on artificial intelligence-A European approach to excellence and trust	19/02/2020	The document gives a definition of AI, underlining it’s benefits and technological advances in different areas, including medicine, security, farming, as well as identifying it’s potential risks: opaque decision making, gender inequality, discrimination, lack of privacy, bias, etc.	No	Yes	No
Data quality and artificial intelligence – mitigating bias and error to protect fundamental rights	11/06/2019	Algorithms used in machine learning systems and artificial intelligence (AI) can only be as good as the data used for their development. High quality data are essential for high quality algorithms. Yet, the call for high quality data in discussions around AI often remains without any further specifications and guidance as to what this actually means.	No	Yes	No
Unboxing artificial intelligence: 10 steps to protect human rights	14/05/2019	The document provides a number of steps which national authorities can take to maximize the potential of artificial intelligence systems and prevent or mitigate the negative impact they may have on people’s lives and rights. It focuses on 10 key areas of action.	No	Yes	No
Ethics guidelines for trustworthy AI (HLEG)	08/04/2019	The Guidelines put forward a set of 7 key requirements that AI systems should meet in order to be deemed trustworthy.	Yes	Yes	No
Understanding algorithmic decision-making: opportunities and challenges	05/03/2019	The expected benefits of algorithmic decision systems (ADS) may be offset by the variety of risks for individuals (discrimination, unfair practices, loss of autonomy, etc.), the economy (unfair practices, limited access to markets, etc.) and society as a whole (manipulation, threat to democracy, etc.). They present existing options to reduce the risks related to ADS and explain their limitations. They sketch some recommendations to overcome these limitations to be able to benefit from the tremendous possibilities of ADS while limiting the risks related to their use. Beyond providing an up-to-date and systematic review of the situation, the report gives a precise definition of a number of key terms and an analysis of their differences. The main focus of the report is the technical aspects of ADS. However, other legal, ethical and social dimensions are considered to broaden the discussion.	No	Yes	No
Preventing unlawful profiling today and in the future: a guide	05/12/2018	This guide explains what profiling is, the legal frameworks that regulate it, and why conducting profiling lawfully is both necessary to comply with fundamental rights and crucial for effective policing and border management. The guide also provides practical guidance on how to avoid unlawful profiling in law enforcement agencies and border management operations.	No	Yes	No
BigData: discrimination in data-supported decision making	30/05/2018	This focus paper specifically deals with discrimination, a fundamental rights area particularly affected by technological developments.	No	Yes	No
European AI strategy	25/04/2018	Aims at making the EU a world-class hub for AI and ensuring that AI is human-centric and trustworthy.	No	Yes	No
Fundamental rights implications of big data	14/03/2017	The text considers the potential use of big data in both commercial and law enforcement areas, as well as the risks, particularly in terms of unlawful discrimination and bias. It also emphasises the need for greater algorithmic accountability and transparency, calling on the commission and member states to ensure, with appropriate guidelines, that data-driven technologies do not jeopardize the exercise of fundamental rights.	No	Yes	No
General data protection Regulation (GDPR)	27/04/2016	The general data protection regulation (GDPR) protects individuals when their data is being processed by the private sector and most of the public sector. The processing of data by the relevant authorities for law-enforcement purposes is subject to the data protection law enforcement directive (LED) instead. No mention of biases.	No	No	Yes

### 3.3 Similarities and differences in definitions of algorithmic bias

With regard to the definition of the phenomenon of algorithmic bias, only four guidelines provide an explicit definition (see Supplementary Appendix I and Annex I). In particular, this section discusses the similarities and differences between the definitions given in (1) “Bias in Algorithms – Artificial Intelligence and Discrimination” (FRA, 2022), (2) “Algorithmic discrimination in Europe” (European Commission, 2021c), (3) “Assessment List for Trustworthy AI” (ALTAI) (AI HLEG, 2020), and “Ethics Guidelines for Trustworthy AI” (AI HLEG, 2019). All definitions exhibit commonalities as they acknowledge that algorithmic bias within AI systems has the capacity to result in unjust or discriminatory outcomes. Whether it takes the form of differential treatment rooted in protected characteristics, systematic errors, or instances of unfairness, there is a shared consensus concerning the potential adverse effects. Additionally, there is unanimous agreement across these definitions that bias in AI can originate from a multitude of sources encompassing data handling, algorithm design, and societal norms. This collective recognition underscores the intricate and multifaceted nature of the issue at hand. Furthermore, each of these definitions acknowledges that algorithmic bias is not confined to a mere technical interpretation but rather embraces a multidimensional concept that necessitates consideration of various facets, ranging from the technical intricacies involved to the ethical implications it carries.

Differences among these definitions become evident when considering their respective emphases. The definition found in “Bias in Algorithms – Artificial Intelligence and Discrimination (FRA, 2022)” places primary focus on the legal and normative dimensions of bias, with a particular emphasis on discrimination and bias-motivated crimes, distinguishing it from the others that encompass a more extensive range of technical and ethical considerations. “Algorithmic discrimination in Europe” (European Commission, 2021c) introduces a notable distinction between general systematic errors and those specifically tied to fairness, a subtle nuance absent from the remaining definitions, thereby underscoring the significance of fairness as a distinct facet within the realm of algorithmic bias. On the other hand, the definitions offered by “Assessment List for Trustworthy AI (ALTAI) (AI HLEG, 2020)” and “Ethics Guidelines for Trustworthy AI (AI HLEG, 2019)” accentuate the diversity of AI platforms and systems in which bias may emerge, implying a broader applicability than the initial two definitions. This expansive viewpoint acknowledges that bias can manifest across an array of AI contexts and systems, emphasising its multifaceted presence in the AI landscape.

In summary, while these definitions of algorithmic bias share common ground in recognising its negative consequences and diverse sources, they also exhibit differences in focus, nuance and breadth of application. These differences reflect the multidisciplinary nature of the concept and the need to address it from different angles, including legal and ethical considerations as well as technical aspects.

### 3.4 Recommendations to mitigate/minimize algorithmic bias

Finally, this review presents an organized compilation of mitigation/minimisation measures extracted from the 86% of the analyzed guidelines (see section 3.2). These measures are intended to serve as recommendations for effectively addressing discriminatory biases within AI systems (see Supplementary Appendix I and Annex II)^6^. To facilitate clarity and comprehensiveness, the measures identified in this review have been categorized into three excluding categories depending on the stage of development of the AI systems. It provides a holistic and structured approach to understanding and evaluating AI systems. An AI system, as any technology, has to be created, managed and used. This structured approach ensures that all critical aspects of AI technology lifecycle are covered, promoting a balanced view that can inform policy and decision-making:

1. Design: in terms of technical designing issues of the AI system. Focusing on design helps to identify points where interventions can prevent ethical issues before they arise.
2. Governance: during the internal management of the development of the AI system. This category is fundamental as it addresses the external controls and standards that shape the AI landscape, ensuring compliance with societal values and legal requirements.
3. Organizational: with regard to the implementation and monitoring of the AI system. This category is essential for studying the real-world impacts of AI in work environments and societal contexts, providing a bridge between theoretical design and practical application.

The total number of measures considered in this compilation is 152, encompassing a comprehensive set of approaches for mitigating bias in AI. The mean total score across all categories is 7.24^7^ (SD = 4.38). The measures in the compilation exhibit a wide range, with scores ranging from 0 to 14 in a single document, reflecting the diversity and complexity of bias mitigation strategies in AI contexts. Table 3 summarises the quantitative results of the compilation of bias mitigation/minimization measures.

**Table 3:** Description of quantitative results of bias mitigation measures compilation.

Type of measure	*N*	%	*M*	SD	Min	Max
Design	55	36	2.62	2.04	0	8
Governance	63	42	3.00	2.47	0	7
Organizational	34	22	1.62	1.72	0	7
Total	152	100	7.24	4.38	0	14

**Figure 2:**
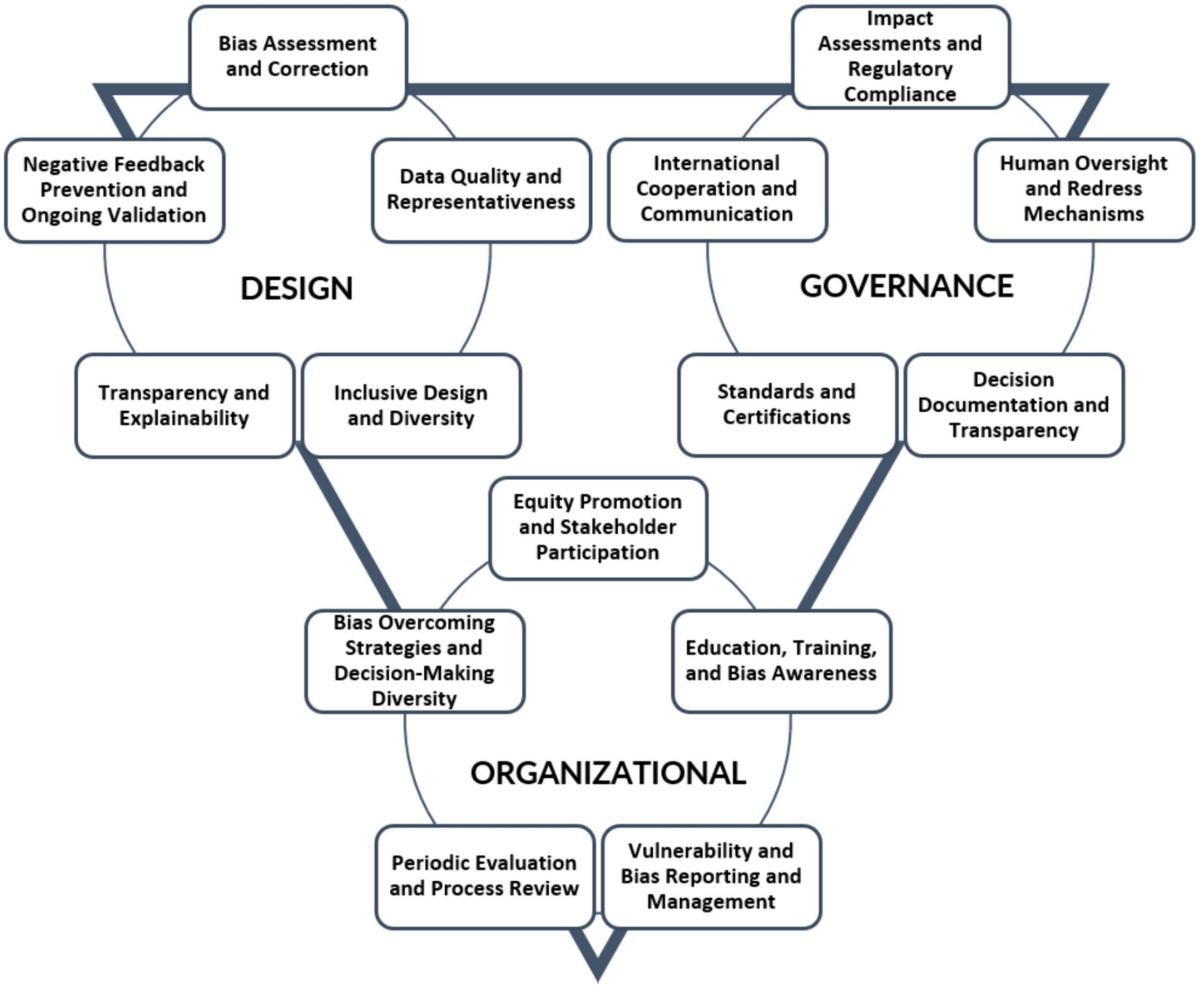
Categories of bias minimization measures from the European regulatory framework of AI.

In order to summarize the bias minimization/mitigation measures presented in Supplementary Appendix I and Annex II in a practical way, Figure 2 presents the excluding subcategories that have been established from the original recommendations:

#### 3.4.1 Design measures

55 measures have been identified in this category, accounting for 36% of the total. The mean score for Design measures is 2.62 (SD = 2.04). The measures in this category range from a minimum score of 0 to a maximum of 8.

- Bias assessment and correction: directly address the identification and rectification of biases within AI systems. It involves pre-deployment testing for biases, ongoing monitoring, and the implementation of algorithmic adjustments to mitigate identified biases.
- Data quality and representativeness: ensure that the datasets used for training AI systems are accurate, comprehensive, and reflective of the diversity of the target population. This includes the collection of high-quality data, the assessment of data sources for representativeness, and the elimination of data that may introduce or perpetuate bias.
- Inclusive design and diversity: inclusion of a wide range of linguistic, cultural, and demographic characteristics in the development of AI systems. It promotes the creation of tools and models that can understand and process diverse forms of natural language and cater to a broad user base.
- Transparency and explainability: develop AI systems’ ability to provide clear, understandable explanations for its decisions and actions. This includes the development of interpretable models, the documentation of algorithmic processes, and the communication of AIsystem capabilities and limitations to users.
- Avoidance of feedback loops and ongoing validation: implement mechanisms to prevent AI systems from perpetuating and reinforcing their own biases over time, often referred to as “feedback loops.” it also involves the continuous validation of AI systems to ensure they are performing as intended and without discriminatory effects.

#### 3.4.2 Governance measures

This category comprises 63 measures, making up 42% of the total. The mean score for Governance measures is 3.00 (SD = 2.47). The range of scores for Governance measures spans from 0 to 7.

- Impact assessments and regulatory compliance: conduct fundamental rights impact assessments and ensure compliance with regulations such as GDPR and existing laws and regulations. They ensure that AI systems are developed and deployed in compliance with legal standards.
- Human oversight and redress mechanisms: ensure human involvement in the oversight of AI systems. It also includes the establishment of mechanisms for individuals to seek redress if they are adversely affected by an AI system.
- Decision-making transparency: record and share key decisions made during the development and deployment of AI systems. They aim to create an audit trail that can be reviewed to ensure ethical and regulatory compliance.
- Standards and certifications: adopt and adhere to industry standards and certifications that guarantee the quality and ethical integrity of datasets and AI mechanisms.
- International cooperation and communication: share best practices, research findings, and policy approaches across international borders. It promotes collaboration among nations, organizations, and stakeholders in the field of AI to establish common standards.

#### 3.4.3 Organizational measures

34 measures have been identified in the Organizational category, representing 22% of the total. The mean score for Organizational measures is 1.62 (SD = 1.72). Organizational measures have scores ranging from a minimum of 0 to a maximum of 7.

- Equity promotion and stakeholder participation: promote fairness and equity in AI systems and encourage the involvement of diverse stakeholders throughout the AI lifecycle, from design to deployment and evaluation.
- Education, training, and bias awareness: develop educational programs and training initiatives for AI designers and developers on recognizing and managing biases or potential biases.
- Vulnerability and bias reporting and management: establish protocols for reporting and managing potential vulnerabilities and biases in AI systems. This includes the creation of channels through which internal staff and external parties can report concerns.
- Periodic evaluation and process review: implement regular assessments to ensure data accuracy and representativeness and that AI systems processes continue to function without biases and with accuracy.
- Bias overcoming strategies and decision-making diversity: develop strategies to handle biases and ensure diversity in decision-making teams, reducing the risk of homogenous biased outcomes.

## 4 Discussion and conclusion

Throughout the previous sections that make up this work, different aspects related to the European normative-ethics response to the algorithmic biases in the context of AI systems have been described. Our study adds significant added value in a number of ways to the state-of-the-art. Overall, the study combines for the first time a European scope and a comprehensive and analytical review of authoritative documents of different types and sources, making it a valuable resource for advancing the understanding and management of discriminatory bias in AI systems. In general terms, the problems surrounding this issue are quite clear with regards to the potential negative impacts of biases on certain segments of the population, such as those represented by certain ethnic groups, gender or race. As can be seen from the European guidelines analyzed, there is a general trend that systematically disadvantages these groups due to various factors such as faulty data collection, possible biases that the designers of the tools may unconsciously transfer to the algorithm and, finally, the uses that are made of these tools in different contexts. However, there is a heterogeneity of methodologies and definitions when addressing the problem of bias, its impact and its mitigation/minimisation measures. This highlights at least two things: firstly, that there is no clear conceptual framework on the issue at the European level; secondly, that this lack of a clear conceptual framework may affect the concreteness and detail of the possible mitigation/minimisation measures proposed. In other words, without a common set of key definitions, there may be a lack of clarity and appropriateness of the mitigation/minimisation measures to be applied. For example, a lack of consensus on what constitutes fair or unfair discrimination or a disproportionate impact on a population group. As discussed at the beginning of this paper with reference to the existence of bias and its consequences, the existence of disproportionate impact is not always synonymous with unfair discrimination. For example, a recruitment algorithm that prioritises certain technical skills may disproportionately exclude groups that have historically had less access to technical education, without implying a direct discriminatory intent.

This problem of a common frame of reference in terms of available methodologies and definitions results in a plurality of solutions which are not always effective and which may be altered depending on the initial defining conditions, which can generally lead to the non-intertranslatability of solutions dedicated to the mitigation/minimization of biases and which may pose an additional problem for the developers of AI tools when it comes to establishing the ethical/legal action criteria to be followed in order to comply with European regulations and standards. In addition to this problem, we can also find others related to the measurement and quantitative and qualitative assessment of biases and their possible impact. The absence of standard metrics for analysis and the contextual, multidimensional and evolutionary dynamics of biases translate into a greater effort to try to establish measures to mitigate and minimize them. Finally, although they are not the subject of analysis in this paper, it is also worth pointing out technical limitations in terms of being able to make the measures described here effective. In general terms, we can find those related to the amount of data collected and available and their subsequent impact on the efficiency, accuracy and possible social impact of the tools. If the amount of data available is not sufficiently representative, there may be a loss of accuracy in the operationalization of the tools or other problems related to affecting under-represented (or over-represented) groups. However, this may also be due to the ethical, legal and political challenges of dealing with particular population groups or implementing mitigation measures in the face of possible biases. Finding the right balance between different aspects is therefore a considerable challenge that has not yet been solved.In any case, in response to the results of this paper, there is a need for intensification of efforts in some very interesting lines of research. First, the continuous assessment of a unified European conceptual framework for addressing AI biases, including standard definitions and methodologies (i.e., AI Act). Second, conducting global comparative studies to identify best practices and areas for improvement. Third, advancing technologies to mitigate bias in AI, with a focus on robust and fair algorithms. In addition, studying the specific impact of AI bias in different sectors, such as healthcare and criminal justice, to understand its impact on different populations. Educating and raising awareness of AI biases among developers, policymakers and the public is also crucial. In addition, fair data collection and analysis methods should be explored to minimize inherent biases, establishing methodologies for regular ethical and social impact assessments of AI systems (beyond ALTAI), with a focus on bias identification and management. These research avenues could significantly improve the understanding and management of discriminatory bias in AI, both in Europe and globally. Lastly, it’s also worth noting limitations to the study conducted here, mainly of a methodological nature: (a) the lack of a detailed analysis of sources that do not reference the European normative framework,^8^ which could be perceived as a lack of plurality; (b) the analysis of technical literature addressing the issue of algorithmic biases and their possible mitigation/minimization and impact; (c) the fact that it is not systematic implies the likelihood of missing some relevant documents, therefore its reproducibility cannot be guaranteed. However, the impact and relevance of this study compared to a systematic review is ensured by the last limitation, which is of a substantive nature, common to any study in the field of AI: the dynamic evolution of the issue of algorithmic biases is leading to the rapid proliferation of a large literature and, at the same time, the obsolescence of the older on.

## Author contributions

PC-M: Conceptualization, Formal analysis, Investigation, Methodology, Supervision, Validation, Writing – original draft, Writing – review & editing. AN-S: Formal analysis, Investigation, Methodology, Supervision, Validation, Visualization, Writing – review & editing. FC-T: Formal analysis, Methodology, Resources, Supervision, Validation, Writing – review & editing.

## Footnotes

^1^More details about AI Act are available in the web of the European Commission: *AI Act. Shaping Europe’s digital future. AI Act* (European Commission, n.d.).

^2^To find out more about the European approach to AI, see also: *Facing the future. AI-driven research projects overcoming real-life challenges. Research and Artificial Intelligence* (European Commission, n.d.).

^3^However, it should be noted that there is a diversity of opinions regarding the discriminatory potential of AI tools when used in recruitment-related contexts. Without denying their possible negative impacts, it does seem necessary to point out those researches that point to their positive aspects. In particular, several research have also pointed out the potential of the AI tools to mitigate human bias in recruiting process (Houser, 2019). In addition, it has also been stated that AI tools in comparison with human decision-making can improve efficiency, objectivity and consistency of the decisions (Kassir et al., 2022).

^4^This problem can be seen in Table II and Annex I, where only 4 documents out of 21 contain definitions of the term “algorithmic bias.”

^5^In this sense, it should be noted that the use of the term bias and its meanings refers to the phenomenon and, on the other hand, the term “discrimination” in its different meanings refers to the negative consequences that the use of tools that operate with biased AI models can cause. On the other hand, it is also necessary to highlight that while AI tools may contain biases (as humans do), not all of them necessarily lead to potentially negative consequences for individuals.

^6^It is important to note that the proposed categories of measures to minimise or mitigate discriminatory bias (i.e. design, governance and organisational) come from the analytical structure and definitions provided internally in the STARLIGHT project for the same purpose. In this sense, any criticism of the arbitrary nature of these definitions is justified, as a completely different approach could have been taken. From a methodological point of view, we would also like to point out that although only one of the authors made a preliminary classification of the different measures contained in the documents analysed, it was only in subsequent internal meetings that all the authors agreed on (1) whether a measure belonged in one category or another and (2) the aggregation of measures. Due to the implementation of this procedure, it was decided not to utilize interrater-reliability measures such as Fleiss’ Kappa, Intraclass Correlation (ICC), and Kendall’s Coefficient of Concordance. This decision was based on the alignment of the implemented procedure with our project’s specific needs, which offered a more practical approach for our analysis. In any case, we agree that this could be considered a methodological limitation of our proposal, but not relevant from an informative and decision-oriented perspective to see previous versions of the IA ACT also see (European Parliament, 2023) and European Commission (2021b).

^7^The average number of measures per document in total is indicated here.

^8^In this sense, one of the possible limitations of this study is the absence of a more detailed treatment of the specific actions that the different member states are taking through their national regulations to mitigate or avoid the potential negative impacts that could derive from bias and algorithmic discrimination.
