# Supplementary material for: Analyzing the European institutional response to ethical and regulatory challenges of artificial intelligence in addressing discriminatory bias
Source: Front Artif Intell. 2024 Jun 25;7:1393259. doi: 10.3389/frai.2024.1393259 (PMC11231082; doi:10.3389/frai.2024.1393259)
Supplement: Supplementary file 1 [file Table_1.docx]

Supplementary Materials

# Supplementary Figures and Tables

**ANNEX I. Definitions of algorithmic bias from European AI regulatory framework**

| **Reference** | **Definitions** |
| --- | --- |
| Bias in Algorithms – Artificial Intelligence and Discrimination (2022)  (pp.22—24) | The term ‘bias’ can have a different meaning depending on the context in which it is used and the particular discipline it comes from, for example law or computer science.  It is therefore important to clarify its meaning in the context of this report. Bias can refer to any of the following.  ― Differential treatment based on protected characteristics, such as discrimination and bias-motivated crimes. This refers to an inclination for or against a person or group based on protected characteristics, such as ethnic origin, gender, religion, colour or sexual orientation. Discrimination defines a situation in which an individual is disadvantaged in some way on the basis of ‘one or multiple protected grounds’. Crimes committed with a bias motivation are a particularly severe example of a result of biases against people based on their (assumed) characteristics. Such definitions are often used in legal contexts and the social sciences.  ― Differentiation. Bias understood in this sense is necessary for the proper functioning of a statistical or machine learning algorithm. For example, a machine learning model that has to differentiate between oranges and pears has to have bias towards labelling round, orange objects as oranges. Such use of bias is mainly found in computer science and machine learning.  ― Statistical bias. This refers to the systematic difference between an estimated parameter and its true value. Statistical bias exists when data are not adequately measuring what they are intended to measure. For example, gross domestic product per capita is not necessarily a good measure of the standard of living in a country, as it does not account for inequality of income distribution. In addition, data and the resulting statistical estimates may not be representative of the target population. For example, if a sample of the general population contains more men than women, it is said to be biased towards men. Bias is mainly understood in this way in statistics.  ― Offset from origin. In the context of deep learning, bias is also the name for an estimated parameter. The fixed number indicating the average baseline estimate in the linear weight functions of neural networks is called bias; it is often referred to as a ‘constant term’ or ‘intercept’ in classical regression analysis. It is a purely technical term, and as such it is not relevant to the present discussions, although it is used in neural networks.  Bias is analysed in the context of discrimination (as a legal and normative concept) in this report. Discrimination is mainly linked to prejudices picked up or enshrined in data, but may also be the result of statistical bias. |
| Algorithmic discrimination in Europe. Challenges and opportunities for gender equality and non-discrimination law (2021) (pp..47-48) | Specifically, ‘algorithmic bias’ refers to ‘a systematic error’ of any kind in the outcome of algorithmic operations. Bias therefore has a much wider meaning than discrimination as it is not only concerned with unfair errors but with all kinds of ‘systematic’ errors, which can include those of a statistical, cognitive, societal, structural or institutional nature. When invoked in the particular context of ‘fairness’, however, ‘algorithmic bias’ refers to a particular type of error that ‘places privileged groups at a systematic advantage and unprivileged groups at a systematic disadvantage’. This definition shares commonalities with the legal definition of discrimination understood as the differential unfavourable treatment of an individual or group or the disproportionately disadvantageous impact of a given measure or policy on a specific group. However, the term ‘algorithmic bias’ is more encompassing than the legal term ‘algorithmic discrimination’ as it refers to any kind of disadvantage that could be viewed as ethically or morally wrong. For example, an algorithm that disadvantages low-income groups and privileges people with high incomes could be seen as entailing a form of algorithmic bias from an ethical point of view. From a legal point of view, however, algorithmic discrimination only pertains to the unjustified unfavourable treatment of, or disadvantage experienced by, specific categories of population protected by the law either explicitly (e.g. protected grounds) or implicitly (e.g. general or open-textured non-discrimination clauses). For example, in the context of EU gender equality and non-discrimination law, algorithmic discrimination refers to discrimination based on one of the six grounds explicitly listed in and protected under Article 19 TFEU, that is sex, race or ethnic origin, disability, sexual orientation, religion or belief and age. This is why the term ‘algorithmic discrimination’ will be used throughout this report to refer to the types of algorithmic bias that are problematic from the point of view of EU gender equality and non-discrimination law. |
| Assessment List for Trustworthy AI (ALTAI) (2020)  (p.23) | AI (or algorithmic) bias describes systematic and repeatable errors in a computer system that create unfair outcomes, such as favouring one arbitrary group of users over others. Bias can emerge due to many factors, including but not limited to the design of the algorithm or the unintended or unanticipated use or decisions relating to the way data is coded, collected, selected or used to train the algorithm. Bias can enter into algorithmic systems as a result of pre-existing cultural, social, or institutional expectations; because of technical limitations of their design; or by being used in unanticipated contexts or by audiences who are not considered in the software's initial design. AI bias is found across platforms, including but not limited to search engine results and social media platforms, and can have impacts ranging from inadvertent privacy violations to reinforcing social biases of race, gender, sexuality, and ethnicity. |
| Ethics Guidelines for Trustworthy AI (HLEG) (2019)  (p.36) | Bias is an inclination of prejudice towards or against a person, object, or position. Bias can arise in many ways in AI systems. For example, in data-drive AI systems, such as those produced through machine learning, bias in data collection and training can result in an AI system demonstrating bias. In logic-based AI, such as rule-based systems, bias can arise due to how a knowledge engineer might view the rules that apply in a particular setting. Bias can also arise due to online learning and adaptation through interaction. It can also arise through personalisation whereby users are presented with recommendations or information feeds that are tailored to the user’s tastes. It does not necessarily relate to human bias or human-driven data collection. It can arise, for example, through the limited contexts in which a system in used, in which case there is no opportunity to generalise it to other contexts. Bias can be good or bad, intentional or unintentional. In certain cases, bias can result in discriminatory and/or unfair outcomes, indicated in this document as unfair bias. |

**ANNEX II. Bias mitigation measures raised in European AI related documents**

| **Reference** | **Year** | **Type of measure** | **Specific minimisation/mitigation measures** |
| --- | --- | --- | --- |
| ARTIFICIAL INTELLIGENCE ACT (AI Act)    (p. 57, 61,70, 90, 203, 213, 215) | 2021 / 2024 | Design | 1. Include for example governance arrangements in that specific context of use, such as arrangements for human oversight according to the instructions of use or, complaint handling and redress procedures, as they could be instrumental in mitigating risks to fundamental rights in concrete use-cases. 2. In order to protect the right of others from the discrimination that might result from the bias in AI systems, the providers should, exceptionally, to the extent that it is strictly necessary for the purpose of ensuring bias detection and correction in relation to the high-risk AI systems, subject to appropriate safeguards for the fundamental rights and freedoms of natural persons and following the application of all applicable conditions laid down under this Regulation in addition to the conditions laid down in Regulations (EU) 2016/679 and (EU) 2018/1725 and Directive (EU) 2016/680, be able to process also special categories of personal data, as a matter of substantial public interest within the meaning of Article 9(2), point (g) of Regulation (EU) 2016/679 and Article 10(2), point (g) of Regulation (EU) 2018/1725. 3. Examination in view of possible biases that are likely to affect the health and safety of persons, have a negative impact on fundamental rights or lead to discrimination prohibited under Union law, especially where data outputs influence inputs for future operations. 4. The bias detection and correction cannot be effectively fulfilled by processing other data, including synthetic or anonymised data. 5. Develop technical robustness for high-risk rsystems. They should be resilient in relation to harmful or otherwise undesirable behaviour that may result from limitations within the systems or the environment in which the systems operate (e.g. errors, faults, inconsistencies, unexpected situations). 6. High-risk AI systems that continue to learn after being placed on the market or put into service shall be developed in such a way as to eliminate or reduce as far as possible the risk of possibly biased outputs influencing input for future operations (‘feedback loops’), and as to ensure that any such feedback loops are duly addressed with appropriate mitigation measures. |
|  |  | Governance | 1. Developing a strategy for regulatory compliance, including compliance with conformity assessment procedures and procedures for the management of modifications to the high-risk AI system; 2. Classify as high-risk, insofar as their use is permitted under relevant Union and national law, a number of AI systems intended to be used in the law enforcement context where accuracy, reliability and transparency is particularly important to avoid adverse impacts, retain public trust and ensure accountability and effective redress 3. Qualify as high-risk AI systems intended to be used by a judicial authority or on its behalf 4. Create codes of conduct. |
|  |  | Organizational | 1. Remain aware of the possible tendency of automatically relying or over-relying on the output produced by a high-risk AI system (‘automation bias’), in particular for high-risk AI systems used to provide information or recommendations for decisions to be taken by natural persons; 2. Design and develop appropriate technical solutions to prevent or minimise harmful or otherwise undesirable behaviour |
| BIAS IN ALGORITHMS: ARTIFICIAL INTELLIGENCE AND DISCRIMINATION    (pp. 7-15, 77-78) | 2022 | Design | 1. Test for bias before and regularly after deployment. 2. Provide guidance on when and how to collect and safeguard data on sensitive attributes and how to assess training data quality (in order to avoid “feedback loops”). 3. Promote language diversity in tools available for natural language processing. |
|  |  | Governance | 1. Decide when an algorithm cannot be used and should be abandoned. 2. Assess ethnic and gender biases, highlighting potential under- and over-flagging of content. 3. Share the information necessary to assess bias with relevant oversight bodies (equality bodies and data protection authorities, which should employ specialised staff and cooperate with data protection authorities and other relevant oversight bodies). 4. Increase knowledge, awareness and resources for bias testing of algorithms (increase access to resources needed for evidence-based oversight of algorithms, share data and data infrastructures…). |
|  |  | Organizational | 1. Assess outputs, specially on particular groups or areas with little research. |
| AUDITING THE QUALITY OF DATASETS USED IN ALGORITHMIC DECISION-MAKING SYSTEMS    (pp. II-III, 16-40) | 2022 | Design | 1. Adopt a preventing approach (using techniques that correct biases in AI systems from the first stages of the AI tool development process, via pre-processing, in-processing, and post-processing). 2. Differentiate between patterns in the data that represent factual knowledge that we want the AI-based system to learn (e.g., obesity increases colorectal cancer risk) and stereotypes that we want to avoid (e.g., fat people do not have exercise habits). 3. Create or use high quality domain-specific training datasets (ensure that training, validation and testing data sets are sufficiently relevant and representative), and continuously assess the quality and integrity of the data. |
|  |  | Governance | 1. Include the 'human in the loop' during the development process and build diverse, interdisciplinary development teams with ethical reflection and inclusive participation. 2. Consider the GDPR concept of “fairness”, and apply DPIAS and IA impact assessments. 3. Adopt standards and certificates applicable to datasets and AI mechanisms, both in terms of the information to be included in a dataset and the types of procedures that will ensure the absence of bias in an IA system. 4. Monitor high-risk AI tools and delimitation of uses according to the assigned risk (through adequate tools, such as dynamic monitoring and providing citizens and NGOs with tools to complain or sue). |
|  |  | Organizational | 1. Strengthen AI-system-subject transparency rights to find the source of biased results. |
| ETHICS BY DESIGN AND ETHICS OF USE APPROACHES FOR ARTIFICIAL INTELLIGENCE    (pp. 3-21) | 2021 | Design | 1. Specify the steps which will be taken to ensure data about people is representative of the target population and reflects their diversity or is sufficiently neutral (document how bias in input data and in the algorithmic design will be identified and avoided). Establish a formal process to guarantee the selection of data for the system will be fair, accurate and unbiased (initial assessment, auditable mechanisms…). It should be assumed that any data gathered is biased, skewed or incomplete until proven otherwise. |
|  |  | Governance | 1. Incorporate ethical principles into the development process. 2. "Data minimisation and data protection should never be leveraged to hide bias or avoid accountability, and these should be addressed without harming privacy rights”. 3. Fair impacts: ensure that the AI system does not affect the interests of relevant groups in a negative way, and document methods to identify and mitigate negative social impacts in the medium and longer term. 4. Transparency: address all the relevant ethical issues, such as the removal of bias from a dataset, and keep records of all relevant decisions to allow tracing how ethical requirements have been met. 5. Universal accessibility: design AI systems to be usable by different types of end-users with different abilities. |
|  |  | Organizational | 1. Guarantee that both internal staff and third parties can report potential vulnerabilities, risks or biases, and are aware of the limits of the system. |
| ALGORITHM DISCRIMINATION IN EUROPE. CHALLENGES AND OPPORTUNITIES FOR GENDER EQUALITY AND NON-DISCRIMINATION LAW  (pp. 11, 140-151) | 2021 | Design | 1. Include preventive strategies in the design, training and development phases of the creation of algorithms (equality impact assessments and equality by design strategies offering guidance on the equality law framework to computer and data scientists). 2. Implement technological debiasing strategies to minimise algorithmic discrimination both at the level of data selection, labelling and use, and at the level of algorithmic models themselves. 3. Intervene ex post through the use of screening and auditing algorithms that can detect discrimination. 4. Use open and clean data for training and control purposes. |
|  |  | Governance | 1. Create dedicated monitoring and supervising institutions, both public (EU equality body) and private that promote the use of non-discriminatory algorithms. 2. Create soft-law instruments such as ethical codes, self-regulation practices such as voluntary codes of conduct, recommendations and guidelines, cooperation between data protection agencies and equality bodies and the setting up of public-private alliances. 3. Adopt the draft Horizontal Directive under negotiation at the council since 2008 and an expansive interpretation of the personal scope of EU equality law. 4. Continuously monitor and test high-risk algorithms and their output, set up auditing, labelling and certification mechanisms, and encourage watchdogs and whistleblowers to signal suspicions of algorithmic discrimination. 5. Promote a better representation of all minority groups in the professional communities designing and training algorithms to favour a diversity of perspectives (gender equality, among others). 6. Include active human involvement: human-centred AI or human-in-the-loop systems designed to avoid rubber-stamping, complemented by supervision and consultation mechanisms (chain of control and consultation with users), with a clear allocation of liability and legal responsibility. 7. Facilitate legal redress by increasing transparency (e.g. open data requirements for monitoring purposes, such as access to source codes), explainability and accountability, and by combining different legal tools to foster clear attribution of legal responsibilities, clear remedies, fair rules of evidence, flexible and responsive interpretation and application of non-discrimination concepts. |
|  |  | Organizational | 1. Raise awareness, train and educate about the risks of algorithmic discrimination linked to the use of AI and ways to tackle it among IT specialists but also all relevant professional communities (regulators, judges, economic players and the society at large). |
| GETTING THE FUTURE RIGHT. ARTIFICIAL INTELLIGENCE AND FUNDAMENTAL RIGHTS | 2020 | Design | N/A |
|  |  | Governance |  |
|  |  | Organizational |  |
| PRESIDENCY CONCLUSIONES – THE CHARTER OF FUNDAMENTAL RIGHTS IN THE CONTEXT OF ARTIFICIAL INTELLIGENCE AND DIGITAL CHANGE    (pp. 5-14) | 2020 | Design | 1. Data used to train AI systems have to be accurate and adequate for their purpose, and potential biases have to be addressed. |
|  |  | Governance | 1. Address opacity, complexity, bias, a certain degree of unpredictability and partially autonomous behaviour to ensure the compatibility of automated systems with fundamental rights and to facilitate the enforcement of legal rules. 2. Adopt a human-centric and fundamental rights based approach. 3. Pay special attention to marginalised individuals and groups and those in vulnerable situations. 4. Make public participation easier and more effective. 5. Data protection rules and other legal and ethical norms need to be ensured and appropriate safeguards have to be in place, specially in sensitive matters (mass surveillance, facial recognition systems, hate speech in online platforms…). 6. Make AI systems transparent and explicable. |
|  |  | Organizational | 1. Ensure that decisions based on algorithmic systems are less prone to biased results than human-made decisions, and allow better-targeted individual assistance and treatments, benefitting the whole social community and promoting the social protection and healthcare of vulnerable groups. |
| ASSESSMENT LIST FOR TRUSTWORTHY ARTIFICIAL INTELLIGENCE (ALTAI)    (pp. 5, 16-18, 22) | 2020 | Design | 1. Avoid creating or reinforcing historic unfair bias from the data. 2. Consider diversity and representativeness of data (test for specific target groups or problematic uses). 3. Guarantee mechanisms to ensure fairness in your AI system, and a quantitative analysis or metrics to measure and test the applied definition of fairness. 4. Create a mechanism that allows for the flagging of issues related to bias, discrimination or poor performance of the AI system. |
|  |  | Governance | 1. Perform a prior fundamental rights impact assessment to check whether it potentially negatively discriminates against people (testing and monitoring during development, deployment and use phases, and rectifying measures). 2. Enable inclusion and diversity throughout the entire AI system’s life cycle. 3. Make sure AI systems are user-centric and designed in a way that allows all people to use AI products or services, regardless of their age, gender, abilities or characteristics. 4. Assess and put in place processes to test and monitor for potential biases during the entire lifecycle of the AI system. 5. Set educational and awareness initiatives to help AI designers and AI developers be more aware of the possible bias they can inject in designing and developing the AI system. 6. Identify the subjects that could potentially be (in)directly affected by the AI system, and consult with the impacted communities or groups. 7. Consult stakeholders (solicit regular feedback even after deployment and long term participation). |
|  |  | Organizational | 1. Establish clear steps and ways of communicating on how and to whom bias issues can be raised. Establish a process for third parties (e.g. suppliers, end-users, subjects, distributors/vendors or workers) to report potential vulnerabilities, risks or biases in the AI system. |
| RECOMMENDATION CM/REC(2020)1 OF THE COMMITTEE OF MINISTERS TO MEMBER STATES ON THE HUMAN RIGHTS IMPACTS OF ALGORITHMIC SYSTEMS  (pp. 7-8, 12-13) | 2020 | Design | 1. Assess quality of datasets in algorithmic systems, considering human rights and non-discrimination rules that may be affected as a result of the quality of the data that are being put into and extracted from an algorithmic system. Attention should be given to the provenance, shortcomings, and the possibility of inappropriate or decontextualized use of the dataset. Be aware of risks related to the quality, nature, and origin of data used for training their systems, ensuring that errors, bias, and potential discrimination in datasets and models are addressed within the specific context. 2. Ensure that the functioning of the algorithmic systems is tested and evaluated with due regard to the fact that outputs vary according to the specific context in which they are deployed and the size and nature of the dataset that was used to train the system, including with regard to bias and discriminatory outputs. 3. Ensure that testing on personal data of individuals is performed with diverse and sufficiently representative sample populations, ensuring that relevant demographic groups are neither over- nor under-represented, and not draw on or discriminate against any particular demographic group. |
|  |  | Governance | 1. Identify and/or develop appropriate institutional and regulatory frameworks and standards that set benchmarks and safeguards to ensure the compatibility of the design, development and ongoing deployment of algorithmic systems with human rights. 2. Invest in relevant expertise to be available in adequately resourced regulatory and supervisory authorities. 3. Regular testing and continuous evaluation, reporting and auditing against state-of-the-art standards related to completeness, relevance, privacy, data protection, other human rights, unjustified discriminatory impacts and security breaches before, during and after production and deployment, to detect technical errors, legal, social, and ethical impacts. 4. Ensure that the staff involved has sufficiently diverse backgrounds to avoid deliberate or unintentional bias. 5. Follow a standard framework for human rights due diligence to avoid fostering or entrenching discrimination throughout all life-cycles of their systems. Seek to ensure that the design, development, and deployment of their systems do not have direct or indirect discriminatory effects on individuals or groups that are affected by these systems, including on those who have special needs or disabilities or who may face structural inequalities in their access to human rights. |
|  |  | Organizational | 1. Foster democratic participation and general public awareness of the capacity, power and consequential impacts of algorithmic systems. 2. Ensure that the development of algorithmic systems is discontinued if testing or deployment involves the externalisation of risks or costs to specific individuals, groups, populations and their environments. 3. Stop the development of algorithmic systems if human rights impact assessments or testing phases identify significant risks or negative externalities that cannot be mitigated. |
| THE ETHICS OF ARTIFICIAL INTELLIGENCE: ISUES AND INITIATIVES    (pp. 2, 16, 30-36, 47) | 2020 | Design | 1. Develop a fairness definition, define what a fair outcome looks like, and include that in the development process. 2. Assume that biases exist within data and thus within systems built from these data, and strive not to replicate them. 3. Search for training data representative of the task and the different groups. |
|  |  | Governance | 1. Allow the communication about the possible existence of biases. 2. Ensure fairness and transparency through being able to know why an automated program made a particular decision: explainable systems, intentional understanding (through validation, investigation and evaluation of the program during development), and algorithm auditors. 3. Accountability: respect the regulation; and Control: “human in the loop”, and “the big red button”. 4. Minimise the “black box” nature of machine learning, through codes of conduct and initiatives to spot biases earlier. |
|  |  | Organizational | N/A |
| GENDER EQUALITY STRATEGY 2020-2025 | 2020 | Design | N/A |
|  |  | Governance |  |
|  |  | Organizational |  |
| WHITE PAPER ON ARTIFICIAL INTELLIGENCE    (pp. 11-15, 18-24) | 2020 | Design | 1. Avoid faulty and biased training data at the design stage, and create mechanisms to ensure that quality of data is maintained throughout the use of AI. 2. Follow specific requirements and control for certain particular AI applications (remote biometric identification). |
|  |  | Governance | 1. Record the process of data selection, keeping of the data, and documentation on programming, training methodologies and techniques avoiding biases. 2. Human oversight: monitoring, intervention and validation of the outcomes, so that it does not lead to outcomes entailing prohibited discrimination. 3. Enable prior conformity assessments and enhance compliance with legal requirements (and its enforcement). 4. Encourage international cooperation. 5. Inform about the capabilities and limitations of the AI system, and against the “black box effect”, both for citizens and researchers. |
|  |  | Organizational | N/A |
| DATA QUALITY AND ARTIFICIAL INTELLIGENCE - MITIGATING BIAS AND ERROR TO PROTECT FUNDAMENTAL RIGHTS  (p.3, pp.8-9, pp.11-13) | 2019 | Design | 1. Establish a constant assessment to ensure the quality of the data through the following measures: the study of possible errors in the data such as lack of precision, representativeness of the samples of the data collected. 2. Use of the concepts of reliability and validity in the collection and processing of the data to be used. 3. Elaboration of detailed descriptions of the data sets to be used in order to be able to know their contents and to guarantee their quality. 4. Ask questions such as:   -What information is included in the data?  -Is the information included in the data appropriate for the purpose of the algorithm?  -Who is covered in the data?  -Who is under-represented in the data?  -What is the time frame and geographical coverage of the data collection used for building the application? |
|  |  | Governance | N/A |
|  |  | Organizational | N/A |
| UNBOXING ARTIFICIAL INTELLIGENCE: 10 STEPS TO PROTECT HUMAN RIGHTS  (pp. 12-15) | 2019 | Design | 1. Process the data in a proportionate manner in relation to the legitimate purpose pursued by such processing, and shall reflect at all stages of the processing a fair balance between the interests pursued by the development and deployment of the IA system and the rights and freedoms at stake. |
|  |  | Governance | N/A |
|  |  | Organizational | 1. Introduce a legislative framework providing adequate safeguards where AI systems are based on the processing of genetic data; personal data relating to criminal offences, criminal proceedings and convictions, and related security measures; biometric data; personal data relating to 'racial' or ethnic origin, political opinions, trade union membership, religious or other beliefs, health or sex life. These safeguards should also provide protection against discriminatory or biased processing of these data. 2. Promotion of AI literacy. |
| ETHICS GUIDELINES FOR TRUSTWORTHY AI  (p.12, pp.17-18, p.27-30, p. 29) | 2019 | Design | 1. Ensure the quality and integrity of the datasets that are collected, processed and subsequently used in AI tools. 2. Avoid unfair biases caused by the use of incorrect, outdated or inaccurate data. 3. Establish monitoring and follow-up measures at different stages of the life cycle of AI tools. |
|  |  | Governance | 1. Guarantee the principle of equity through a fair and equal distribution of benefits and costs, ensuring that individuals and groups are not unfairly biased. 2. Ensure inclusion and diversity throughout the lifecycle of AI systems, encouraging participation and ensuring equal access through inclusive design processes. Seek regular feedback even after the deployment of AI systems and establish mechanisms for long-term stakeholder involvement. 3. Ensure accessibility and universal design, so that systems are user-centred, user-friendly and socially responsive. 4. Introduce mechanisms to enable others to report potential problems related to the existence of bias. |
|  |  | Organizational | 1. Guarantee the principle of equity through a fair and equal distribution of benefits and costs, ensuring that individuals and groups are not unfairly biased. 2. Encourage stakeholder participation in developing and auditing AI systems. 3. Communicate potential or perceived risks such as those related to the possible existence of bias. 4. Introduce processes for workers or external parties to report potential vulnerabilities or biases in the IA system or its application. |
| UNDERSTANDING ALGORITHM DECISION-MAKING: OPPORTUNITIES AND CHALLENGES  (pp. I-VII, p.25, 41, pp. 66-67, p.76) | 2019 | Design | 1. Promotion of the principle of fairness. 2. Utilization of certifications and labels in order to enhance the trust in algorithmic decisions systems. 3. Ensure appropriate creation of datasets. 4. Be aware about possible technical constrains. 5. Avoidance/Mitigation of opacity in AI tools. 6. Re-train data constantly. 7. Pre-processing possible existing bias. 8. Give the possibility to test systems across numerous domains and via numerous methodologies. |
|  |  | Governance | 1. Ensure adequate measures in order to avoid non-discrimination. 2. Give the possibility to test systems across numerous domains and via numerous methodologies. |
|  |  | Organizational | 1. Promotion of the principle of fairness. 2. Utilization of certifications and labels in order to enhance the trust in algorithmic decisions systems. 3. Avoidance/Mitigation of opacity in AI tools. |
| PREVENTING UNLAWFUL PROFILING TODAY AND IN THE FUTURE: A GUIDE  (pp.11-12, p. 22,48,60,72, pp.80-81) | 2018 | Design | 1. Use reliable data based on accuracy, quality or representativeness. 2. Algorithmic profiling that is legitimate, necessary and proportionate. 3. Knowledge of fundamental rights and their application in their given context. |
|  |  | Governance | 1. Be aware of fundamental rights and their application in their given context. 2. Conduct assessments to find out whether there are norms and practices that perpetuate explicit or implicit prejudices and negative stereotypes. 3. Ensure that performance indicators are linked to the prevention of prejudice and stereotypes. 4. Introduce specific courses and/or training sessions focusing on addressing personal and institutional bias and stereotypes. |
|  |  | Organizational | 1. Inform individuals by providing them with information about the data to be collected, stored and processed. 2. Be aware of fundamental rights and their application in their given context. 3. Reflect on whether their decision is justified by objective information in order to avoid unlawful or biased profiling. 4. Provide timely and detailed information to officers, for example in 'pre-shift briefings' at the beginning of each shift in order to guide officers on how to conduct their duties. 5. Conduct assessments to find out whether there are norms and practices that perpetuate explicit or implicit biases and negative stereotypes. 6. Introduce specific training courses and/or sessions focused on addressing personal and institutional biases and stereotypes. 7. Ensure that performance indicators are linked to the prevention of prejudice and stereotypes. |
| BIGDATA: DISCRIMINATION IN DATA-SUPPORTED DECISION MAKING  (p.5, 8,11) | 2018 | Design | 1. Highlight the importance of data quality and its potential to affect unfair biases 2. Exclude information about protected groups such as gender or ethnicity from the dataset. 3. Check whether protected characteristics of individuals can be inferred from other information in the dataset, so-called proxies. 4. Ensure that the way the algorithm was constructed and works can be explained in a meaningful way |
|  |  | Governance | 1. Conduct fundamental rights impact assessments: identify possible biases and abuses in the application and results of algorithms. |
|  |  | Organizational | 1. Conduct fundamental rights impact assessments: identify possible biases and abuses in the application and results of algorithms. 2. Ensure that the way the algorithm was constructed and works can be meaningfully explained. |
| EUROPEAN AI STRATEGY  (p.14,16) | 2018 | Design | 1. Develop AI systems in a way that allows humans to understand (the basis for) their actions. |
|  |  | Governance | 1. Supporting research into the development of explainable AI. |
|  |  | Organizational | 1. Develop AI systems in a way that allows humans to understand (the basis for) their actions. 2. Supporting research into the development of explainable AI. |
| FUNDAMENTAL RIGHTS IMPLICATIONS OF BIG DATA  (Articles, 20,21,22; statement M) | 2017 | Design | 1. Establish procedures that can ensure data quality and avoid biased algorithms, spurious correlations, errors or underestimation of legal, social and ethical implications. |
|  |  | Governance | N/A |
|  |  | Organizational | 1. Establish periodic assessments of the representativeness of data sets, consider whether they are affected by biased elements, and develop strategies to overcome such biases. 2. Review the accuracy and significance of data analysis predictions on the basis of impartiality and ethical concerns. 3. Assess the need not only for algorithmic transparency, but also for transparency about possible biases in the training data used to make inferences based on big data. |
| GENERAL DATA PROTECTION REGULATION (GDPR) | 2016 | Design | N/A |
|  |  | Governance |  |
|  |  | Organizational |  |
